# Spectacle dispensing for myopia at primary eye care level

**Published:** 2019-05-13

**Authors:** Sathya T Ravilla, Dhivya Ramasamy

**Affiliations:** 1 Medical Officer: Paediatric Ophthalmology and Strabismus, Aravind Eye Hospital, Madurai, India.; 2 Senior Faculty: Lions Aravind Institute of Community Ophthalmology, Aravind Eye Care System, Madurai, India.

**Primary eye care services must include refractive error assessment and spectacle dispensing.**

**Figure:**
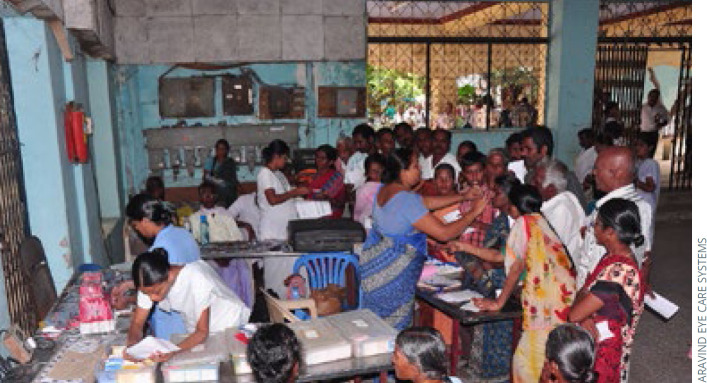
Spectacle dispensing at a rural Aravind eye camp. An optician helps patients choose from a selection of frames while technicians select the prescribed lenses, edge and fit them. INDIA

The prevalence of myopia has been progressively increasing^1^,^2^,^3^ and this is due to changes in lifestyle or reduced outdoor activity. Myopia is easily corrected with a pair of spectacles. However, lack of access to refractive assessment and availability of spectacles remain the key challenge in addressing uncorrected refractive error.^4^ Primary eye care services are best positioned to create and sustain such accessibility. Well-designed primary eye care can provide the required refractive correction for the community.

These services need to be comprehensive and include not only refractive assessment and prescription, but also, spectacle dispensing. It has been noted that making spectacles available on the spot is important to ensure uptake and use.^5^

This article discusses guidelines for how myopia correction can be provided at the community level through outreach camps and primary care centres. To make spectacle dispensing available, we need to have systems in place to provide the right inventory of lenses and the right kind of frames stocked to ensure uptake and patient satisfaction.

## Guidelines for prescribing spectacles

Spectacles need to be prescribed based on the needs and symptoms. Most practitioners prescribe spectacles for a refractive error of −0.7 dioptres (D) and less only if the patient is symptomatic.^6^ Spectacles should be prescribed based on the patient's subjective refraction and not merely retinoscopy findings. Those with presbyopia may be given the option to remove their myopia spectacles for reading or use bifocals.

Cycloplegic refraction is recommended when prescribing spectacles for the first time especially in children less than 15 years of age. The AAO paediatric ophthalmology panel recommends prescribing spectacles for myopia of:

- 5.0 D or more in infants
- 4.0 D or more in children between one to two years of age
- 3.0 D or more in those between two to three years of age and
- 2.5 D or more in children over three years of age.^7^

Spectacles need to be prescribed for even lower refractive errors in those with anisometropia.

## Guidelines for dispensing spectacles

While dispensing spectacles, it is important to take into account the patients need, vocation and socio-economic background. Opticians need to guide patients to choose appropriate frames. Frames that are too large can slip down the nasal bridge and could disturb the alignment of optic centre of the lenses to the patients' pupil. In younger children, plastic frames and plastic lenses are recommended to avoid injury to the eye if the spectacles break.

- Plastic lens (CR 39) has the advantage of being safe and more durable. However, the edging and fitting of these lenses need more sophisticated equipment which is neither suitable for portable use nor feasible for a small scale of operations. Also, as these lenses tend to yellow over time, they cannot be stocked for long periods. Plastic lenses are available with additional features such as anti-reflection, UV protection, scratch resistance, high refractive index lenses etc.
- Glass lenses are cheaper, easier to process and are less prone to scratches; although they are heavier and can break more easily. Glass lenses are used for eye camps as they are less expensive and can be edged by hand using a portable edging machine.
- For patients with high myopia, lenses with a high refractive index are thinner and give a better cosmetic result. They are available both in plastic and glass and are best if dispensed with anti-reflection coating.
- Polycarbonate lenses are recommended for children for safety but they are more expensive; these are especially recommended for those who are one-eyed and need spectacles for protection.

## Spectacle dispensing in outreach camps

Outreach camps are designed to include a refraction assessment as part of the standard clinical examination. On-the-spot dispensing of spectacles at the campsite ensures uptake and use of spectacles. Often 15–25 per cent of the patients at a camp will require spectacles and the lens inventory stock must be planned accordingly and at affordable prices. Refraction camps conducted at workplaces are a good way to address uncorrected refractive error in the working age group. As the number of patients who require spectacles is around 35 per cent in these camps, a larger inventory needs to be planned.

Patients are offered a choice of spectacle frames to choose from. A standard inventory of ready-made lenses has been developed for different sized camps. Ready-made lenses in common power ranges are easily available in the market. Usually this can cater to about 90 per cent of the prescriptions. Lenses for high powers, mixed astigmatism and hyperopic astigmatism are rarely required and hence not stocked – these are made to order against a prescription and couriered to the patient. This arrangement can ensure an increased spectacle uptake of about 80 per cent among those who are given prescriptions.^9^

## Spectacle dispensing at primary eye care centres

Primary eye care services must include refractive error assessment and spectacle dispensing. A simple way to provide spectacles is to offer a range of spectacle frames and outsource edging and fitting of the lenses. At Aravind Eye Care System in India, a network of primary eye care centres or vision centres (VCs) are linked to the central spectacle processing unit at the base hospital.

These VCs are managed by two vision technicians. Patients receive a comprehensive eye examination including refractive evaluation and consult with an ophthalmologist using telemedicine. Each VC carries a standard display of about 80 frames of varying colours, models and sizes, besides a small inventory of reading glasses. An online ordering system conveys the choice of frame, lens type and prescription details to the central spectacle processing unit. This allows the patient to have the choice of ordering plastic lenses. Spectacles are delivered to the patient within one to three days. This ensures over 90 per cent uptake of spectacles and spectacle sales contribute over 60 per cent of these centres' income.

**Figure:**
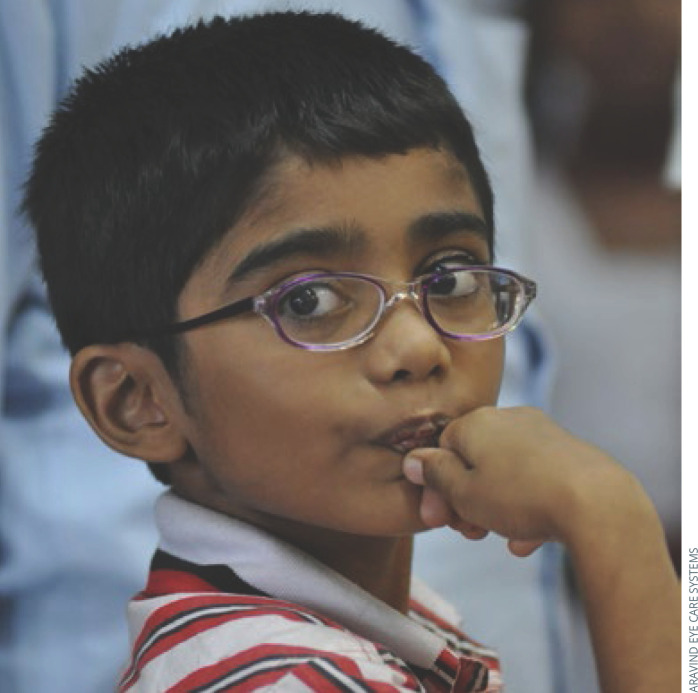
The right frame will ensure proper alignment of the optic centre of the lens to the patient's pupil. INDIA

**What do you need to dispense spectacles at an outreach camp?**

*For an average of 200 camp patients*

- 30–40 patients expected to be prescribed spectacles
- Inventory:
  - Presbyopic glasses: 25
  - Frames: 80
  - Lenses: 300 (varying powers)
- Human resources: two opticians for sales and fitting
- Equipment: lens markers, chipper, cutter, lens edger, screwdrivers, frame warmer, adjustment pliers
- Percentage of on the spot delivery: 85 per cent
